# Attitudes and Perceptions of Suicide and Suicide Prevention Messages for Asian Americans

**DOI:** 10.3390/bs5040547

**Published:** 2015-12-04

**Authors:** Priyata Thapa, Yoonhee Sung, David A. Klingbeil, Chih-Yuan Steven Lee, Bonnie Klimes-Dougan

**Affiliations:** 1 Teachers College, Columbia University, New York, NY 10031, USA; E-Mail: pt2379@tc.columbia.edu; 2 Richard Oaks Counseling Center, Richardson, TX 75081, USA; E-Mail: yoonheesungphd@gmail.com; 3 Department of Educational Psychology, University of Wisconsin-Milwaukee, Milwaukee, WI 53201, USA; E-Mail: davidak5@uwm.edu; 4 Family and Child Studies, Montclair State University, Montclair, NJ 07043, USA; E-Mail: leech@mail.montclair.edu; 5 Department of Psychology, University of Minnesota, Minneapolis, MN 55455, USA

**Keywords:** Asian American (AA), suicide prevention, public service announcements

## Abstract

Understanding the context of suicidal behaviors is critical for effective suicide prevention strategies. Although suicide is an important topic for Asian Americans, there is limited information about what Asian Americans’ attitudes are towards suicide and their perceptions about the effectiveness of prevention efforts. These questions are critical to examine to provide foundational knowledge for determining how best to intervene. In this study, Asian American (*n* = 87) and White (*n* = 87) participants completed self-report indexes on their knowledge of depression and suicide (e.g., estimates of suicide rates), coping attitudes (e.g., help-seeking) and suicide prevention attitudes (e.g., usefulness of PSAs). The results indicate that in comparison to Whites, Asian Americans perceived suicidal behavior to be more common, perceived a stronger link between depression and suicide, less frequently endorsed help-seeking strategies, and reported more concern or distress after viewing a suicide prevention PSA. These preliminary results also suggest the possibility of cultural differences in perceptions of suicide prevention messages. The implications of these findings are discussed with a focus on providing recommendations for exploring suicide prevention efforts for Asian Americans.

## 1. Perception of Suicide and Suicide Prevention Messages for Asian Americans

Suicide is a leading cause of death among adolescents and young adults and is one of the most pressing public health issues across much of the world. Suicides in Asian countries constitute about 60% of suicides in the world (Wei and Chua) [1], with Sri Lanka, Japan and South Korea representing the Asian countries having the highest rates of suicide (Hendin *et al.*) [2]. By contrast, in the U.S., there is a considerable variation across minority populations with regard to suicide risk (Goldston *et al.* [3]; Joe, Canetto and Romer [4]; Walker, Townley and Asiamah [5]). The suicide rate among the Asian American and Pacific Islander population was 5.40 per 100,000 in the U.S. This rate is approximately half of the rate for the U.S. overall of 10.75 per 100,000 (CDC, 2007, as cited in Lizardi and Gearing [6]). Additionally, there has been a rising concern about suicidal behaviors (which include suicidal preoccupation and suicide attempts) observed among Asian Americans (Chung [7]), particularly among Asian American young adults (Cutler, Glaeser and Norberg [8]; Hijioka and Wong [9]). Despite the relatively low rate of deaths in Asian Americans, preventing suicidal behavior and suicide deaths in this minority group remains an important objective. Our understanding is limited concerning how Asian Americans perceive suicide risk and suicide prevention efforts. These questions are important to examine as they provide foundational knowledge to consider how best to intervene. 

## 2. Perceptions of Mental Illness and Suicide in Asian Cultures

The term “Asian American” (AA) includes over 16 ethnic groups and reflects more than twice as many languages (Lai and Arguelles, 2003, as cited in Duldulao, Takeuchi and Hong [10]). Among these diverse ethnicities is an array of cultural and religious values that promote a variety of beliefs about the consequences of suicide (Bhugra [11]; Nelson, Hanna, Houri and Klimes-Dougan [12]; Vijayakumar *et al.* [13]). Nevertheless, there are several commonalities in beliefs, attitudes, and behaviors among Asians living in Asia and AAs. Therefore, also, there are likely some commonalities among AAs who have ancestors from different regions of the East. For instance, since the majority of Chinese individuals in the U.S. are foreign-born or raised by those who are foreign-born, their overall behaviors reflect strong Chinese cultural values, beliefs, behaviors and practices (Ma [14]), which may influence their general perception of circumstances and their solutions, including their mental well-being and suicidal behaviors. 

Eastern traditions may undergird many of the perceptions about suicide for AAs. In contrast to the West, where individualism is highly regarded, those in the East commonly adhere to collectivist values, promoting harmonious interactions, cooperation and conformity to others’ expectations (e.g., Markus and Kityama [15]). In the East, significant pressures for vocational, financial or social success are commonly experienced (e.g., Sue, Sue, Sue and Takeuchi [16]). Individuals who perceive that they have failed to live up to these standards may consequently believe that they have dishonored or brought shame to their families and communities. For adolescents and young adults, these pressures are often associated with academic achievement, and these pressures are particularly high in Asian cultures (Stipek [17]). The pressure to succeed and the shame that results from the failure to do so, which are associated with psychological distress and suicide, have been documented among youth in Asian countries, such as Sri Lanka, China and Japan (Lee, Anderson and Klimes-Dougan, in press [18]; Turner, Husman and Schallert [19]; Vijayakumar, Pirkis and Whiteford [20]; Vijayakumar *et al.* [13]).

Asian Americans may hold other beliefs that may in turn influence their attitudes and perceptions about suicide risk and prevention. In many Eastern cultures, depression and other forms of mental illness are viewed as a sign of personal weakness that brings shame to the family (Tzeng and Lipson [21]). Suicidal behaviors among AA youths are thought to be under-recognized (Goldston *et al.* [3]), in part because AAs are often less emotionally expressive, have greater difficulties discussing problems with their family members and do not disclose suicidal ideation as readily as their White counterparts (Komiya, Good and Sherrod [22]; Morrison and Downey [23]; Rhee, Chang and Rhee [24]). For example, South Asian communities tend to discourage the open expression of emotions and emphasize shyness, restraint and subordinacy, particularly for females (Marecek [25]).

There are also ways in which the perceptions of the mind-body relationship impact suicidal thought and behavior. It is common for AAs to view physical and psychological problems as being strongly connected and to believe that emotional disturbances are led by physical problems (Sue and Sue [26]). Additionally, fatalism, the belief that life is determined by external forces (Peck and Bharadwaj [27]), is consistent with some Eastern values (e.g., Kar, Alcalay and Alex [28]; Lassiter [29]) and may influence perceptions of suffering and suicide (e.g., Jamieson and Romer [30]). These different fundamental belief systems may influence how suicidal behaviors are perceived and labeled across different ethnic groups (Cauce *et al.* [31]).

These perceptions may also take into account that certain factors that precipitate suicidal behavior may be stronger in Asian countries, compared to the West, including acute life stresses and consequent stress reactions (Beautrais [32]). A study conducted on hospitalized suicide attempters in Singapore concluded that interpersonal problems were the most common risk factors (Mak, Ho, Chua and Ho [33]). In China and India, suicide appears to be weakly linked to depression and more strongly linked to acute life stressors (Wei and Chua [1]). In some South Asian countries with higher rates of suicide, suicides are commonly preceded by family abuse, arguments and conflicts with intimates and in-laws, and unreciprocated love (Chua [34]; Halliburton [35]; Marecek [25]; Widger [36]). Suicide also results as a response to intolerable circumstances by individuals with no better alternatives, to violation of one’s rights, or to contest the rigidity of prejudices and inequities within the social system, including social class and gender status (Staples and Widger [37]; Widger [38]). Some regions in Asia have also experienced increasing suicide rates with the rapid sociocultural change or the repercussions of not being able to find jobs suited for higher educational achievement (Halliburton [35]). In Asia, protective factors for suicide may be both common to other regions of the world (e.g., feeling emotionally supported), as well as somewhat unique (e.g., feeling expression of regret; Mak, Ho, Chua and Ho [33]).

Asians who immigrate to the U.S. may face additional challenges associated with immigration, minority status, acculturation, and ongoing intergenerational conflicts that may influence their perceptions of suicide and suicide prevention (Hwang and Goto [39]; Lau, Jernewall, Zane and Myers [40]). Many AA youths experience confusion with dual identities (Rho and Rho [41]), which may be distressing to them. However, the association between perceptions of suicide, suicidal behavior and minority status may not always be straightforward. For example, Duldulao *et al.* [10] found that immigrants were at lower risk for suicidal behaviors in comparison to their U.S.-born counterparts. In their study, U.S.-born AA women showed the most suicidal behaviors. It is possible that the stigmatization of suicide decreases as AA immigrants become more acculturated, thus increasing the risk of suicidal behaviors.

Finally, religious beliefs undoubtedly shape the way individuals derive meaning from their life and their views about death and an afterlife. As such, religion likely plays an important role in suicidal behaviors and perceptions of suicide in AAs. Although some sanctions for suicide associated with Eastern values and religions are highly publicized (e.g., hara-kiri, sati, suicide bombings), many beliefs are likely to serve as a protective function by providing high values of life (e.g., Buddhism) or strong sanctions against killing (e.g., Islam; Nelson *et al.* [12]). Additionally, these broad ethnic differences set the stage for understanding potential methods of coping and help-seeking among AAs.

## 3. Coping and Help-Seeking

The approaches used to seek assistance for ailments are imbedded in one’s cultural perceptions associated with the origins of the problem and beliefs about remedies: some depend on self-reliance and solitary coping mechanisms, such as drinking alcohol or meditating; some turn to their families for emotional support, while some seek help from formal services (Klimes-Dougan, Klingbeil and Meller [42]). For Asians, they may seek the assistance of others by acquiring herbal remedies, acupuncture or the guidance of religious leaders (Ma [14]; Shin [43]). AAs that adhere to collectivist values may adopt specific coping styles and perceptions about suicide (Heppner *et al.* [44]). There is evidence that cultural factors may influence various features of help-seeking, from the identification of a problem to the choice of treatment providers. These views in turn may lead to the differential utilization of mental health services by different ethnic groups for suicide prevention and treatment (Cauce *et al.* [31]). Distinct cultural practices, including coping styles, family ties and religious beliefs, influence people’s willingness to seek help and their ability to respond to mental health services (U.S. Department of Health and Human Services (DHHS) [45]). There is convincing evidence that extremely low levels of treatment seeking for mental health problems distinguish AAs (Leong and Lau [46]) from Whites, African Americans and Hispanic Americans. For example, Loya, Reddy and Hinshaw (2010) [47] found that South Asian college students showed higher reluctance towards the use of counseling services for mental illnesses, and a preference to reject or exclude persons with mental illnesses due to higher relevance of stigma for this population, compared to whites. The underutilization of formal mental health services in AA populations may be attributed to stigma and loss of face (social shame) caused by mental health problems, different traditional explanations for their problems, limited fluency in English (among some immigrants), delays in reaching out (as a result of trying other sources of help first) and the limited availability of culturally-competent mental health professionals (DHHS [45]; Shin [43]). Additionally, the stigma associated with depression and suicidal risk may pose unique challenges for AAs, in that they may be particularly hesitant to seek professional help for their emotional problems (Burr [48]; Fogel and Ford [49]). For example, AAs are often concerned about hampering their social network as a result of their mental health issues, and are thus, discouraged from seeking help (Kim, Sherman, Ko and Taylor [50]; particularly East Asians and East Asian Americans, Goldston *et al.* [3]). Together, these factors suggest that a deeper understanding is needed of the perceptions and attitudes about suicide risk and prevention in AAs.

## 4. Suicide Prevention for Asian Americans

There is limited research on suicide and suicide prevention among AAs (Harachi, Catalano, Kim and Choi [51]) and limited efforts directed to soliciting the views of AAs about what approaches they believe would be useful for intervening. Although the utility of suicide prevention public service announcements (PSAs) have not been extensively evaluated with Asians or AAs, recently, some researchers have suggested that the content of these messages may cause untoward effects (e.g., Chambers *et al.* [52]). For example, when the link between depression and suicide is overemphasized, people might normalize suicide and, as a result, be more likely to consider it a viable option when distressed (Cialdini [53]). Prior suicide prevention studies that assessed the unintended effects associated with PSAs have shown that some high-risk adolescents are less likely to endorse help-seeking strategies and more likely to endorse higher levels of maladaptive coping skills after viewing a billboard suicide prevention message (Klimes-Dougan, Lee and Houri [54]). A similar study involving young adults showed that billboard PSA viewers were less likely to favor help-seeking attitudes, and that high-risk participants tended to overestimate the rates of suicidal ideation and to normalize suicidal thoughts and actions (Klimes-Dougan and Lee [55]). In order to reduce suicidal behaviors in adolescents and young adults, awareness of the interconnectedness of ethnic background, perceptions of suicide, help-seeking attitudes, and impressions of existing suicide prevention efforts is vital for culturally-competent practice, as well as for the development of culturally-sensitive interventions, which are limited at present (Goldston *et al.* [3]).

## 5. Present Study

The purpose of this study is to advance the understanding about suicide in AAs, an ethnic minority group in the U.S. Because most foundational knowledge on this topic to date has been comprised of primarily White participants, a matched comparison group of Whites provides a useful approach to evaluating potential ethnic differences in perceptions of suicide and suicide prevention. In this study, AA and White adolescent and young adult participants were asked to report on their: (1) knowledge of depressive symptoms and perceptions about suicide risk; (2) coping attitudes (e.g., help-seeking) and (3) perceptions about suicide prevention efforts (the focus here was on public service announcements).

## 6. Method and Participants

This study included 174 (67.8% females) study participants who were students attending a high school or college in a metropolitan, Midwestern region of the U.S. Specifically, recruitment of the study participants took place in health classes from one of three high schools (*n* = 40) or from a range of behavioral science courses at a large, public university (*n* = 134). The mean age was 20.4 years (*SD* = 3.9) with a range from 14 to 35 years. A total of 87 participants, who self-identified as AA, were included in this study group. The countries of origin for the AA participants were Korea (18.3%), China (11.4%), Vietnam (6.8%), Laos (6.8%), India (5.7%), Cambodia (4.5%), Thailand (4.5%), Taiwan (3.4%), Philippines (3.4%), Malaysia (2%), Hong Kong (1.1%), Tibet (1.1%), Pacific Islands (1.1%), Iraq (1.1%) and unspecified (27.5%; this item was not included for the high school students, accounting for most of the unspecified cases). Of the AA participants, 44.2% were born in the U.S., and the rest were born abroad. A matched comparison group (based on sex, age, PSA condition, *etc.*) of self-identified White participants was selected from the same high school or university class and condition. See Table 1 for a description of the demographic information for the AA sample and the matched white sample.

**Table 1:** Demographic information for participants.

	Total Sample (*N* = 174)		AA (*n* = 87)		White (*n* = 87)	
**Characteristic**	*N*	%	*n*	%	*n*	%
Sex						
Female	118	67.8	59	67.8	59	67.8
Education						
High School	40	23.2	20	22.9	20	23.3
College	115	65.5	58	66.6	57	66.3
College Plus	16	9.2	8	9.2	8	9.3
Missing	2	1.2	1	1.1	1	1.2
Religion						
Christian	97	56.1	30	34.9	67	77
Jewish	1	0.6	0	0	1	1.1
Eastern Religion	22	12.7	21	24.4	1	1.1
Atheistic/Agnostic	40	23.1	23	26.7	17	19.5
Other	13	7.5	12	14	1	1.1
Birthplace						
U.S.	123	71.5	38	44.2	85	98.8
Other	49	28.5	48	55.8	1	1.2
Depression/Suicide Risk						
At Risk	47	27	24	27.6	23	26.4
	*M*	*SD*	*M*	*SD*	*M*	*SD*
Age	20.4	3.9	20.3	3.8	20.6	4.0

## 7. Procedures and Measures

This study was approved by the Institutional Review Board at a large, public university. Participants who were under the age of 18 years old received both written parental consent and written child assent forms, while adult participants provided written consent for this study. Participants were offered course credit or extra credit for study participation (or for completing an alternative assignment).

The primary goal of the larger study was designed to evaluate the impact of viewing suicide prevention PSAs. Participants in the current study represent a subgroup of the larger study. Namely, all AAs from the larger study were included in this study group, representing 5.7% of the larger study group, which is generally comparable to the proportion of AAs living in this region of the U.S. Approximately half of the participants in this study were also included in one of the previous studies in which the focus was on the impact of viewing a suicide prevention PSA and which did not address the importance of culture (Klimes-Dougan *et al.* [52]; Klimes-Dougan and Lee [53]).

Data collection took place between 2006 and 2011. During the first years of this study, randomization of participants was based on three conditions (exposure to a billboard PSA, a TV advertisement PSA, or no information), while randomization at the later years of this study was based on primarily two conditions (exposure to the initial billboard PSA and an alternative billboard PSA), because a more detailed examination of billboards was warranted since the initial results suggested some untoward effects of the billboard condition (Klimes-Dougan *et al.* [52]). This approach for assigning conditions resulted in the relatively large group of participants who viewed one of the billboards, but since all White participants were matched within class with AA participants, the type of PSA exposure was consistent across ethnic groups.

More specifically, the conditions were as follows: (1) viewing one of two billboard messages, both of which featured a white male adult with the message that suicide can be prevented by treating depression (e.g., “Prevent Suicide, Treat Depression” or the alternative message “Stop Depression from Taking Another Life”), an action step (“See your Doctor”) and a resource (www.save.org) [56] (*n* = 128); (2) viewing a 30-s TV advertisement featuring several adults with a range of ethnicities providing this narrative: “If you think depression is all in a person’s head, you’re right. It’s a brain illness and like other illnesses, it has symptoms. A person suffering from depression might feel sad, empty or numb. They might sleep more or less, eat a lot, or not at all, or show little interest in participating in ordinary activities. Mood swings; including anger and sadness are common. They are often tired, have a hard time concentrating and making decisions. Depression can make those that suffer from it feel hopeless. It can even lead to suicide. If you see the symptoms of depression, get that person to a doctor. With medical help, depression can be treated, and suicide can be prevented. Learn how to stop depression from taking another life. Call SAVE, Suicide Awareness Voices of Education: 1-888-511-SAVE. On the web at save.org” (*n* = 20); or (3) a no information condition (*n* = 26). It is important to note that for this study, we evaluated a universal suicide prevention campaign designed to identify the population at the greatest risk for suicide: middle-aged, white men. However, this study (and the larger study) focused on youth, because youth are thought to be the most susceptible to untoward effects (Gould, Jamieson and Romer [57]).

All participants completed a brief demographic/screening measure that asked questions regarding sex, age, ethnicity, religion and foreign-born status. Participants also reported on their history of depression and suicide (e.g., “Have you felt sad all or most of the time for a period of a month or been depressed within the past year?” or “Have you done something to try to kill yourself in the past year?”) (adapted from the wording of structured diagnostic interviews and screening tool developed by Kroenke, Spitze and Williams [58]).

Finally, participants were asked to complete the Suicide Awareness Questionnaire (SAQ) using scales largely adapted from Gould *et al.* (2004) [59], and Kalafat and Elias (1994) [60]. The SAQ addresses relevant factors, including: (1) knowledge of depression and suicide; (2) coping attitudes; and (3) suicide prevention attitudes.

### 7.1. Depression and Suicide Knowledge

Depression knowledge was based on the percentage of “correct” symptoms of depression that participants could identify, relative to the total number of symptoms listed (Cronbach’s alphas of 0.62 for all participants; 0.61 for AAs and 0.63 for whites). The questions included six “correct” items (e.g., sad or irritable feelings, thoughts of suicide) and five “incorrect” items (e.g., stealing and lying, headaches). 

To evaluate normative perceptions of suicidal thoughts and behaviors, participants were asked to estimate how common it is for people their age to: (1) seriously think about killing themselves (suicidal ideation); (2) try to kill themselves (suicide attempt); and (3) kill themselves (suicide death). Participants also rated how common it is for people who are struggling with depression to commit suicide. Scores reflected estimates of suicidal risk, generally indexes on a 6-point scale ranging from an estimated rate of 0.01% to 50% (e.g., a rating of 6 would indicate participants estimate suicide attempts in 50% or more of their peers).

### 7.2. Coping Attitudes

The Help-Seeking Scale was based on a factor analytically-derived scale with Cronbach’s alpha of 0.60 (Gould *et al.*, 2004) [59]. Participants were asked how they would respond to the question, “What should you do if a friend tells you he/she is thinking about killing himself/herself?” They then rated on a five-point scale (“never” to “always”) the likelihood of engaging in seven help-seeking behaviors (e.g., get advice from another friend, tell my friend to see a mental health professional, talk to an adult about my friend, tell my friend to talk to his or her parents). Cronbach’s alphas for the participants in this study were 0.54 (0.51 for AAs and 0.56 for whites). 

### 7.3. Suicide Prevention Attitudes

Four items were used to assess the perceived utility of PSAs. Participants were asked to rate on a five-point scale (“not at all” to “extremely”) how useful they thought PSAs like a billboard or a television advertisement would be for reminding those struggling with depression to seek help (overall usefulness). The second item asked participants to indicate what type of person they expected would benefit from viewing a PSA like a billboard or a television advertisement about depression and suicide (benefit for whom). Scores were based on the percentage of the six items endorsed. Response choices included a person who is not experiencing depression, a depressed person, a depressed person who is currently thinking about suicide, *etc.* The third item asked participants what type(s) of information they thought may be useful in preventing suicide (what information is useful). Scores were based on the percentage of six items endorsed, and response choices included a brief advertisement on television, a brief advertisement on the radio, advertisements in newspapers or magazines, a billboard, pamphlets at doctor’s offices, *etc.* The fourth item was used to evaluate if the PSAs resulted in feelings of concern and/or distress for those having been exposed to any one of the three PSAs, *i.e.*, the billboard, the alternative billboard and the TV advertisement.

## 8. Results

### 8.1. Preliminary Results

The AA and White groups were not significantly different in terms of age, sex, PSA group assignment or depression risk status. As expected, there were group differences for religion and birthplace (Table 1).

### 8.2. Differences between AAs and Whites

Results reported here use a series of ANOVAs to assess differences between AAs and Whites on: (1) knowledge of depression and suicide; (2) coping attitudes; and (3) suicide prevention attitudes (Table 2). The tenability of the normality and homoscedasticity assumptions were checked prior to conducting the analyses. Although the assumption of homoscedasticity was met, skewness and kurtosis (see Table 2), along with visual analysis suggest that most of the variables were non-normally distributed. However, ANOVA is relatively robust to address departures of normality (e.g., Norman, 2010 [61]). Significant results were adjusted using *p* < 0.005 based on Bonferroni corrections for multiple comparisons. There were no group differences for knowledge of depression or estimates of suicide ideation. However, there was a significant main effect for estimates of suicide attempts, estimates of suicide deaths and estimates of the link between depression and suicide. AAs’ estimates of the incidence of suicide attempts and suicide deaths were higher than that of Whites. The perceived link between depression and suicide was stronger for AAs than for whites. There was a group difference in coping attitudes, although these results failed to reach significance that accounted for multiple comparisons. Specifically, AAs endorsed less help-seeking attitudes than Whites. There was no evidence that any specific features of help-seeking accounted for these differences (no individual items showed group differences).

Suicide prevention attitudes largely failed to differ between the groups. There were no significant differences in perceptions of the overall usefulness of PSAs, who would benefit from seeing a PSA or the type of information that would be useful for a PSA. However, when considering those who viewed a PSA, AAs were more likely to report concern or distress about the information they were exposed to than Whites.

**Table 2:** ANOVAs for participant knowledge and attitudes toward suicide risk and suicide prevention.

	Total Sample (*N* = 174)		AA (*n* = 87)		White (*n* = 87)				
Question	*M*	*SD*	*M*	*SD*	*M*	*SD*	*F*	*df*	*p*
Knowledge of Depression and Suicide Rates									
Depression Symptoms	85%	11%	85%	11%	85%	11%	0.09	1,172	0.760
Suicide Ideation	3.92	1.48	4.05	1.56	3.79	1.38	1.28	1,172	0.260
Suicide Attempts	2.68	1.09	2.91	1.24	2.45	0.87	8.04	1,172	0.005
Suicide Deaths	2.01	1.07	2.28	1.22	1.74	0.82	11.38	1,170	<0.001
Link Depression and Suicide	2.77	1.23	3.10	1.24	2.43	1.13	13.90	1,171	<0.001
Coping Attitudes									
Help-seeking	2.66	0.69	2.54	0.71	2.79	0.65	5.93	1,170	0.016
Suicide Prevention Attitudes									
Overall Usefulness	2.65	0.88	2.69	0.88	2.61	0.89	0.33	1,170	0.564
Benefits for Whom	2.87	1.94	3.01	1.89	2.74	1.99	0.88	1,172	0.351
Type of Information Useful	3.07	1.56	3.07	1.58	3.07	1.56	0.00	1,172	1.00
Concern/Distress *	1.45	0.74	1.52	0.86	1.32	0.58	8.19	1,164	0.005

Note: * based only on participants exposed to a PSA.

### 8.3. Follow-Up Exploratory Analyses

Exploratory analyses considered ethnic group by PSA exposure interactions and factors related to ethnicity (e.g., religion, birth place). The findings failed to yield any evidence suggesting that there was a potential benefit or risk stemming from exposure to a specific type of PSA for AAs for knowledge of depression and suicide, coping attitudes and suicide prevention attitudes; nor did the follow-up analyses yield any significant differences within the AAs for being born in or outside the U.S. or identified religious affiliation (e.g., measured by Judeo-Christian *versus* Eastern *versus* other/no religions). Consistent with evidence in a larger and more culturally-diverse sample (e.g., Klimes-Dougan *et al.* [54]), the analyses found a significant group difference for depression/suicide history (*F*[1,86] = 5.59, *p* = 0.02). Among the AA group, those with a history of depression/suicide (*M* = 2.25; *SD* = 0.90) endorsed fewer adaptive help-seeking attitudes than those without a history of depression/suicide (*M* = 2.65; *SD* = 0.60).

## 9. Discussion

Efforts to tailor suicide preventative interventions to diverse ethnic groups within the U.S. have been limited. The goal of this study was to begin to address this concern by providing some preliminary evidence that documents AAs’ perceptions of suicide and suicide prevention. Our findings suggest key ethnic differences in knowledge and coping attitudes, yielding results that may be useful for shaping prevention efforts.

One of the main findings of this study was that perceptions of suicide risk differed across groups. AAs had higher estimates of the rates of suicide attempts and death by suicide, compared to Whites. Furthermore, AAs perceived the link between depression and suicide to be stronger than Whites. For example, AAs estimated that about 5% of those with depression will die by suicide, while Whites estimated the link between depression and suicide to be about 1%. While documented suicide rates of clinically-depressed youths and adults (e.g., Bostwick and Pankratz [62]) suggest that AAs’ estimates were more accurate, it is unclear if AA participants view suicide as normative and how, if at all, these beliefs will impact behavior in AAs. Indeed, these results are somewhat surprising, given that the link between psychopathology and suicidal behavior is not as strong among some Asian groups. For example, there is evidence that the rate of mental illness in suicide attempters in the Chinese population is much lower, and it is common for suicide attempts in this population to be executed impulsively, with little forethought (e.g., Phillips, Li and Zhang [63]). Nevertheless, it is important not to ignore the possibility that deleterious outcomes may be associated with normative perceptions of suicide for AAs (e.g., Chambers *et al.* [52]). Perceptions that suicide is normative can lead one to believe that suicide is a typical response to life stressors, and that suicide is a common outcome for those who are suffering from depression, and can thus, lower resistance.

The second main finding in this study is that AAs were less likely to endorse help-seeking attitudes than Whites. Many suicidal youths avoid seeking help due to embarrassment, a belief that nobody can help or recognize the problem or a tendency to rely on oneself (Freedenthal and Stiffman [64]). There are a number of reasons relating to collectivist and fatalist values as to why AAs may be less likely to seek help and may be more likely to use problem avoidance and social withdrawal coping strategies (Chang [65]; Goldston *et al.* [3]). Prior research has linked some Asian values or beliefs to help-seeking attitudes that limit utilization of medical and mental health resources among AAs (e.g., DHHS [43]; Ting and Hwang [66]). Seeking professional help increases the risk of exposing the individual’s personal problems to the public and could result in shaming not only the individual, but also their family (Lee and Law [67]; Shen [68]). In addition, shame may also play a significant role in AAs’ tendency of seeking help through informal, personal networks (e.g., family or close friends), instead of formal, professional services (e.g., counselors, therapists, psychologists and doctors) (Yeh, Arora and Wu [69]). While AAs were overall less likely to endorse adaptive help-seeking attitudes when considering a suicidal friend, with a similarly low rate endorsed for both formal and informal sources of help, AAs with a reported history of depression or suicidal behavior were particularly resistant to endorsing common help- seeking attitudes than were AAs without a history of depression or suicide. What was not assessed here was whether these same attitudes about help-seeking when considering a suicidal friend would have been evident, if the AA participants were considering seeking help for themselves; nor would it be warranted to conclude from this study that help-seeking was less adaptive in AAs without including a broader range of culturally-consistent sources of help in the response options, including acquiring herbal remedies, acupuncture, the guidance of religious leaders, *etc.* (Ma [14]; Shin [43]). Yet, the results of this study provide convincing evidence that a better understanding of help-seeking is needed, if suicide prevention efforts are going to optimally meet the needs of AAs.

Additionally, AA participants expressed greater concern and/or distress after viewing PSAs than White participants. The results seem to suggest a more general response to suicide prevention PSA exposure rather than support differences across PSA type (e.g., billboard *versus* TV ad). AAs may be less comfortable thinking about and discussing this topic because they are not used to discussions of suicide and suicide prevention. Suicide discussed in such open and direct terms may, therefore, be particularly disconcerting for this group. This distress experienced may also be due to AAs’ collectivistic values and common experience of shame when violating social standards, such as the prohibition against these types of conversations (Goldston *et al.* [3]; Gudykunst [70]). Discussions of a highly-stigmatized topic may be particularly disconcerting to AAs. For example, Tzeng and Lipson (2004) [21] found that Taiwanese suicide attempters and their families suffered post-attempt stigma on the basis of their cultural beliefs about suicide, such as suicide renders the soul unable to transmigrate or that suicide is inherited. AAs may also be invested in concealing their problems and feel uncomfortable when asked to reflect on suicide more extensively, as was required for this study. Finally, it is possible, as suggested by our previous work with a sample that was primarily White (Klimes-Dougan *et al.* [54]), that for AAs, PSA exposure may primarily elicit feelings of concern for others, rather than concern for self.

This study represents a small, but important step in evaluating perceptions about suicide in AAs. Future efforts are needed to address the limitations of this study. First, the small sample and the heterogeneity of the AAs likely limited the power of this study. These limitations are likely to have particularly impacted the exploratory, within-group analyses (e.g., religion). Second, internal validity may be limited in several regards. Additional scale development (e.g., some constructs on the SAQ relied on single-item scales) and scale validation are needed. Exploratory analyses included constructs yielded from limited information (e.g., place of birth status, religion, depression status). In the future, continued evaluation of the intended effects of PSAs is critical with larger samples, because subtle changes in the message may render different results across cultures. Third, external validity may be limited. The findings of this study may be best generalized to highly-educated AAs, who live in areas where Whites are the majority. Ideally, we would have examined the cultural context and cultural values of AAs. In the future, it will also be important to investigate how these findings correspond to the perceptions of AAs embedded in other regions of the U.S. and across the world (Beautrais [30]), especially given that in this study, the ethnic factors that could significantly influence beliefs about suicide, including country of origin, religion and acculturation, were largely inconclusive (Eshun [71]; Neeleman, Halpern, Leon and Lewis [72]). 

Here, we have limited our evaluation to PSA exposure. Suicide prevention intervention in the U.S. covers a range of interventions, including gatekeeper training, which helps gatekeepers identify at-risk individuals and direct them to appropriate assessment services (Goldsmith, Pellmar, Kleinman and Bunney [73]), school-based prevention programs incorporated into high school curricula to prevent suicidal behavior (Aseltine and Demartino [74]; Garland, Shaffer and Whittle [75]; Zenere and Lazarus [76]), as well as other universal prevention programs (Gould and Kramer [77]). Nevertheless, there are important hints in these findings that may be applied to developing or modifying existing interventions. First, continued evaluation of the stigmas associated with the discussion of suicide should be explored, considering that some of these stigmas may have protective functions, while others may perpetuate maladaptive coping. Furthermore, there is considerable consensus that interventions must be culturally sensitive for them to be maximally effective for individuals belonging to an ethnic minority group (Blanco-Vega, Castro-Olivo and Merrell [78]). Yet, there are differing views as to which approaches might be most useful. Some would argue that tailoring is not recommended for highly-acculturated AAs, while others might argue that tailoring prevention efforts should be directed at each AA cultural group. In addition, although there are few available models for how best to develop or tailor existing interventions for AAs, it may be useful to take the lead from others who have developed or adapted suicide prevention programs for other highly diverse ethnic minority groups. For instance, LaFromboise and Howard-Pitney [79] created and evaluated a life skills development program, called the Zuni Life Skills Development Curriculum, to prevent suicide for many tribes of American Indian youth. Similarly, Allen *et al.* (2006) [80] studied the protective factors found in the Alaska Native community to develop a preventative intervention for this population. Following these strategies that target culturally-relevant protective factors may prove to be beneficial for AAs.

## 10. Conclusions

In conclusion, this study addresses an important area of research that has not been adequately explored. The results of this study identify ethnic differences that would be ideally considered when planning interventions. AAs estimated the risk for suicide to be more prevalent, tended to endorse fewer help-seeking attitudes, and raised more concerns/distress from considering this topic. Together, this profile may limit the benefit they may accrue from available resources, suggesting more is needed to address the needs across a broader range of ethnicities. Most notably, this study highlights the need for more research, so that well-intended efforts to prevent suicide can more optimally serve the desired goals across the U.S. and so that AAs can be more effectively served (e.g., Chambers *et al.* [52]).
